# New Universal Bulk-Fill Composites with Translucency Shift: Impact of Rapid Curing and Ageing on Polymerisation and Mechanical Properties

**DOI:** 10.3390/ma18245613

**Published:** 2025-12-14

**Authors:** Danijela Marovic, Matej Par, Eva Mandic, Tena Smiljanic, Visnja Negovetic Mandic, Vlatko Panduric, Zrinka Tarle

**Affiliations:** University of Zagreb School of Dental Medicine, 10000 Zagreb, Croatia; mpar@sfzg.unizg.hr (M.P.); emandic@sfzg.unizg.hr (E.M.); tsmiljanic@sfzg.unizg.hr (T.S.);

**Keywords:** composite, dental, universal, rapid curing, degree of conversion, flexural strength, flexural modulus, ageing

## Abstract

This study investigated real-time polymerisation kinetics and mechanical properties under accelerated ageing of novel universal bulk-fill composites incorporating reversible addition–fragmentation chain transfer (RAFT) agent ß-allyl sulfone, designed for anterior and posterior applications. Five bulk-fill composites were tested: Tetric Plus Fill and Tetric Plus Flow (new universal composites); their predecessors, Tetric PowerFill and Tetric PowerFlow (Ivoclar); and RAFT-free Ecosite Bulk Fill (DMG). Specimens were polymerised for 3 s (~3000 mW/cm^2^), 10 s (~1200 mW/cm^2^), or 20 s (~1200 mW/cm^2^). Degree of conversion (DC) was monitored during and after curing, with mechanical testing after 24 h and after thermal cycling. DC and maximum polymerisation rate at 4 mm depth were significantly lower than at 0.1 mm for all materials and curing times. Three-second curing accelerated the polymerisation rate at both depths. Except for Ecosite cured for 3 or 10 s as RAFT-free material, DC ratios at 4 mm exceeded 80% of surface values. Tetric Plus Fill and Ecosite exhibited the highest flexural strength after 24 h, while PowerFill and Ecosite showed the highest flexural modulus at 24 h and after thermal cycling. Rapid curing did not compromise mechanical properties after 24 h, except for PowerFlow, the composite with the lowest filler vol%, but negatively affected both flowable composites after ageing. Thermal cycling reduced flexural strength in most tested conditions, but all materials and curing conditions more than satisfied the ISO 4049 requirements. The new simplified universal composite Plus Fill has a higher DC and improved polymerisation kinetics compared to its predecessor, PowerFill. Rapid curing is not recommended for the material without RAFT agents.

## 1. Introduction

Dental bulk-fill composites have become indispensable in modern restorative dentistry, allowing practitioners to restore cavities in increments of up to 4–5 mm, which is twice the thickness achievable with conventional resin composites [1,2]. These materials were developed to overcome the limitations of the incremental layering, such as technique sensitivity, the inclusion of air and impurities between layers, and the time-consuming nature of the procedure. To permit sufficient light penetration up to 4 mm, the composites had to be more translucent, at the expense of their aesthetics [3]. Flowable bulk-fill composites are generally more translucent than sculptable bulk-fills [4,5], but because they must be capped with sculptable composite in the occlusal area for most materials [6], this issue is less critical for the appearance of the restoration.

A new class of universal bulk-fill materials was recently released, claiming the so-called translucency shift, owing to which the materials become opaque at the end of the polymerisation, thus satisfying the aesthetic properties not only in the posterior, but also in the anterior region [7]. Such claims were unmet so far, as the bulk-fill composites were generally considered to be too translucent for anterior use. Even though certain materials already displayed a similar phenomenon, their aesthetics were never sufficient to be used for anterior restorations.

In addition, these new universal bulk-fill composites, Tetric Plus Fill and Tetric Plus Flow (Ivoclar, Schaan, Liechtenstein), are claimed to be able to retain all the crucial properties even when polymerised only for 3 s with a high-irradiance light source [7]. To be able to achieve that, the manufacturers employed several tactics, including the decrease in filler content, adding addition fragmentation chain transfer (AFCT) agent and a Norrish type I photoinitiator Ivocerin [7]. The reduction of filler content is a crucial modification determining the mechanical properties of dental resin composites, especially after exposure to aging [8,9].

Although AFCT technology has been known for some time, the incorporation of thiol-ene-based agents into dental composites was long hindered by their undesirable colour and odour. This challenge was overcome with the introduction of a specially designed β-allyl sulfone additive, first implemented clinically in the bulk-fill composite Tetric PowerFill (Ivoclar) in 2019 [10]. This chemical modification enables the polymerisation of AFCT-containing materials in only 3 s using a light source with very high radiant exitance of 3000 mW/cm^2^ [11,12,13]. Similar experiments in the past, using plasma lamps, resulted in poorly polymerised materials with insufficient polymerisation in subsurface areas [14]. However, the inclusion of the AFCT agent in the composite formulation triggers the phenomenon known as step-like polymerisation. The light-activated radical of the photoinitiator activates monomers, which can undergo either conventional radical polymerisation or chain transfer when they form a single bond with β-allyl sulfone. In the first step, the AFCT agent β-allyl sulfone stops the growth of the methacrylate chain on one side and fragments into a new free radical that can initiate a new chain reaction with methacrylate monomers in the second step. The reaction continues until the neighbouring monomers are consumed or the viscosity of the material prevents further reaction [11,12,13,15]. Chain transfer therefore leads to shorter polymer chains [16,17,18]. Due to the high energy supplied by the curing unit, a higher number of monomers is simultaneously activated, and with the new free radicals released by the AFCT, the polymerisation is more uniform [19,20].

In addition, Tetric PowerFill and its flowable counterpart, Tetric PowerFlow, feature closely matched refractive indices of the filler and the organic matrix, resulting in enhanced translucency before polymerisation. After polymerisation, the refractive index of the polymer becomes higher than that of the monomer, and translucency decreases, which is more aesthetically acceptable [20]. However, despite the reduction in translucency after polymerisation, bulk-fill materials generally remain too translucent for use in cases with high aesthetic demands, such as Class IV restorations [1,21,22].

This translucency shift technology is further advanced in the latest generation of universal bulk-fill materials, Tetric Plus Fill and Tetric Plus Flow. The manufacturer indicates these materials for all cavity classes, including restorations in the anterior segment. It is claimed that these composites undergo a rapid change in translucency, from very translucent to opaque materials with high light scattering at the end of polymerisation. High translucency of unpolymerised material enables deep light penetration and a high degree of conversion (DC) up to 4 mm depth at the start of polymerisation, These new materials also contain AFCT technology, which, according to the manufacturer, enables rapid 3 s curing [7].

All four materials—the predecessors, Tetric PowerFill and Tetric PowerFlow; and the successors, Tetric Plus Fill and Tetric Plus Flow—contain two types of photoinitiators: the conventional photoinitiator camphorquinone, a Norrish type II sensitiser that requires a tertiary amine as a co-initiator for photoactivation, and the alternative diacylgermane-based photoinitiator Ivocerin, a Norrish type I photoinitiator that dissociates into two photoradicals at specific wavelengths [7,13]. Ivocerin therefore effectively promotes rapid 3 s polymerisation by generating more radicals to initiate the polymerisation reaction.

Recent advancements in curing lights have produced devices that use very high intensity to achieve rapid polymerisation. The development of light-curing techniques has been accompanied by technological improvements in light-curing devices, characterised by increased radiant exitance and an emission spectrum within the relevant wavelength range [14,23].

While Tetric PowerFill and Tetric PowerFlow are well-established bulk-fill composites [24,25,26] with documented clinical success, the introduction of successor materials requires careful evaluation to determine whether these new formulations with lower filler content replicate the performance of their predecessors. This is particularly relevant, as the composition has been modified to achieve increased opacity for highly demanding anterior applications. Only a few studies have investigated the DC [10,27] and microhardness [28] of these materials, as well as their mechanical properties after 24 h of ageing [29]. As polymerisation kinetics determine network architecture, shrinkage behaviour, and stress development at the tooth–restoration interface, understanding these processes is essential for interpreting material behaviour and guiding future research into polymerisation shrinkage and stress [30,31,32]. Equally important, the flexural properties of bulk-fill composites, especially when assessed after artificial ageing, are critical predictors of clinical durability, given their correlation with fracture resistance and restoration longevity [33,34]. Therefore, determining whether newly introduced bulk-fill systems can match or exceed the established performance of earlier generations remains central to validating their suitability for routine clinical use.

The aim of this study was to compare the short-term polymerisation kinetics and flexural properties under accelerated ageing of a new class of universal bulk-fill composite materials to their AFCT-containing predecessors. To the authors’ knowledge, this is the first study investigating the influence of aging on the mechanical properties of these materials.

The following hypotheses were set:

- There is a difference in the polymerisation behaviour and flexural properties between the new generation of universal composites, the predecessor composites with AFCT agent, and the reference material.
- Rapid 3 s curing with high radiant exitance leads to a reduction in DC and flexural properties of the tested materials compared to conventional (10 s) and prolonged (20 s) curing.

## 2. Materials and Methods

### 2.1. Materials

In this study, five bulk-fill composite materials were tested: four with AFCT reagent and one reference bulk-fill composite without the AFCT reagent, listed in Table 1.

### 2.2. Methods

#### 2.2.1. Characterisation of the Curing Unit

The curing unit Bluephase PowerCure (Ivoclar) was characterised using the National Institute of Standards and Technology (NIST)-referenced and calibrated spectrometer MARC Light Collector (Bluelight Analytics Inc., Halifax, NS, Canada). Radiant exitance of the light-curing unit was measured at an empty compartment. The radiant exposure at a wavelength range of 360–540 nm was individually collected at a rate of 16 records/s.

The Bluephase PowerCure featured a light guide with an external diameter of 9 mm and an internal diameter of the active emission surface of 8.5 mm. The emission range was 360–540 nm, with wavelength maxima at 410 and 448 nm. The average radiant exitance values (n = 5), measured using the MARC Light Collector spectrometer at the top surface sensor at a 0 mm distance, were as follows:

- 3 s = 3043.8 ± 12.4 mW/cm^2^;
- 10 s = 1188.2 ± 4.1 mW/cm^2^;
- 20 s = 1204.1 ± 3.9 mW/cm^2^.

The total energy emitted during single exposure to each curing protocol in the 360–540 nm range was measured as follows:

- 3 s = 9.1 ± 0.0 J/cm^2^;
- 10 s = 11.9 ± 0.0 J/cm^2^;
- 20 s = 24.1 ± 0.1 J/cm^2^.

#### 2.2.2. Polymerisation Kinetics and Degree of Conversion

Polymerisation kinetics was evaluated using the Fourier-transform infrared (FTIR) spectrometer (Nicolet iS50, Thermo Fisher, Waltham, MA, USA) with an attenuated total reflectance (ATR) accessory.

Three light-curing protocols were used, with Bluephase PowerCure using the following irradiances:

- 3 s protocol: 3 s with 3044 mW/cm^2^;
- 10 s protocol: 10 s with 1188 mW/cm^2^;
- 20 s protocol: 20 s with 1204 mW/cm^2^.

The 20 s protocol represents the ideal curing conditions required by ISO 4049 [35], while the 3 s and 10 s protocols represent curing conditions with similar radiant exposure: a rapid, short period for the 3 s protocol, and a longer period with moderate irradiance for the 10 s protocol.

**Figure 1:**
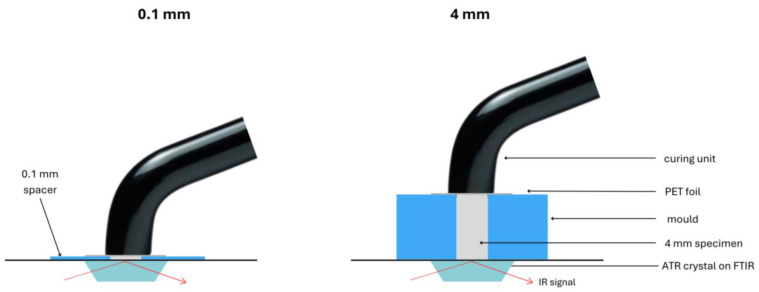
Schematic representation of the experimental setup for polymerisation.

Uncured composites (n = 5 for each material and curing protocol) were placed in a custom-made silicone mould (d = 3, h = 0.1 or 4 mm) covering the ATR diamond and a PET foil on the specimen’s top surface, under the light-curing unit Bluephase PowerCure (Figure 1). Room temperature was kept constant at 21–23 °C. Light curing was activated, and FTIR spectra were taken in real time at a rate of 2 spectra/s for 5 min, with 4 scans and a resolution of 8 cm^−1^ [36].

The changes in the ratio of absorbance intensities of the aliphatic band at 1638 cm^−1^ and aromatic band at 1608 cm^−1^ were used to calculate the DC:

(1) $$\mathrm{DC}\;(\%)=[1-\frac{{\left(1638\;{\mathrm{cm}}^{-1}/1608\;{\mathrm{cm}}^{-1}\right)}_{\mathrm{peak}\mathrm{height}\mathrm{after}\mathrm{curing}}}{{\left(1638\;{\mathrm{cm}}^{-1}/1608\;{\mathrm{cm}}^{-1}\right)}_{\mathrm{peak}\mathrm{height}\mathrm{before}\mathrm{curing}}}]\times 100$$

The DC data were plotted as a function of time, and first derivatives were calculated to represent the reaction rate. The obtained reaction rate was plotted as a function of time to determine the maximum reaction rate (R_max_), described in detail in a previous study [37]. Additionally, the DC values reached at the end of the 5 min observation period (DC_5min_) were evaluated.

A four-parameter exponential sum function fitted the curves of DC versus time.

y = a(1 − e^−bx^) + c(1 − e^−dx^) (2)

The four modulation parameters in this equation are used to describe polymerisation kinetics during the gel phase (parameters a and b) and the glass phase (parameters c and d).

The bottom/top ratio of the DC_5min_ was calculated as the percentage of the values at 4 mm and 0.1 mm:

(3) $$\mathrm{bottom}/\mathrm{top}\;\mathrm{DC}\;\mathrm{ratio}=[\frac{{\mathrm{DC}}_{5\min}\;\mathrm{at}\;4\;\mathrm{mm}}{{\mathrm{DC}}_{5\min}\;\mathrm{at}\;0.1\;\mathrm{mm}}]\times 100$$

### 2.3. Flexural Properties

A total of 600 specimens were prepared, 20 specimens for each material, curing protocol, and aging. Specimens with dimensions 16 mm × 2 mm × 2 mm were made in custom-made Teflon moulds according to NIST 4877 [38]. The split-moulds were positioned on a glass base with the upper and lower surface covered with polyethylene terephthalate (PET) foil and cured with the curing unit Bluephase PowerCure (Ivoclar) in direct contact with the PET foil.

Similar to specimens for the DC, the materials were cured with either of three curing protocols, using a total of six overlapping irradiations, three on the top and three on the bottom side of the specimen:

- “3 s” group—specimens polymerised 6 times for 3 s with radiant exitance of 3044 mW/cm^2^ on both sides.
- “10 s” group—specimens polymerised 6 times for 10 s with radiant exitance of 1188 mW/cm^2^ on both sides.
- “20 s” group—specimens polymerised 6 times for 20 s with radiant exitance of 1204 mW/cm^2^ on both sides.

After polymerisation, each specimen was manually ground with silicon carbide grinding paper (Grit500/P1000, Buehler; Lake Bluff, IL, USA) to remove excess material and assure the uniformity of dimensions of all the specimens. The dimensions of the specimens were checked using a digital calliper (Alpha Professional Tools; Franklin, NJ, USA). After preparation, the specimens were stored in plastic containers filled with 5 mL of distilled water in the dark and in an incubator, at 37 °C.

The specimens underwent two aging conditions:

- 24 h: 24 h of aging in distilled water at 37 °C in the dark.
- TC: 7 days of aging in distilled water at 37 °C in the dark + 10,000 thermocycles (5–55 °C, dwell time 30 s).

All specimens were subjected to a three-point bending test on a universal testing device (Inspekt Duo 5 kN-M, Hegewald & Peschke, Meß- und Prüftechnik GmbH, Nossen, Germany) at a crosshead speed of 1 mm/min. Flexural strength (FS) was calculated according to the following formula:

(4) $$\mathrm{FS}=\frac{3\mathrm{FL}}{{2\mathrm{bh}}^{2}}\;\left[\mathrm{MPa}\right]$$

where F represents the maximum force, L is the distance between support points, b is the width, and h is the height of the specimen.

The flexural modulus (FM) was calculated according to the following formula:

(5) $$\mathrm{FM}=\frac{{\mathrm{FL}}^{3}}{{4\mathrm{bh}}^{3}d}\;\left[\mathrm{GPa}\right]$$

where d represents the deflection of the specimen under the load, F.

### 2.4. Statistical Analysis

An a priori power analysis was performed based on preliminary tests conducted on similar composite materials to determine the sample size required to detect a 10% difference between groups for the primary outcomes, i.e., DC_5min_ and flexural properties. Power analysis calculations were based on a two-tailed comparison with a significance level of 0.05 and statistical power of at least 80%. Under these assumptions, the estimated sample size was n = 5 per experimental group for polymerisation kinetics and n = 20 per experimental group for flexural testing.

A statistical analysis was conducted to assess variations in DC_5min_, R_max_, FS, and FM among five bulk-fill composite materials. For DC_5min_ and R_max_, a three-way ANOVA was performed with the factors “material”, “curing protocol”, and “layer thickness”. When significant interactions between factors were detected, simple main effects were examined by conducting separate one-way ANOVAs for each factor at fixed levels of the other two factors. Pairwise comparisons were adjusted using Tukey’s post hoc procedure. FS and FM were analysed using a three-way ANOVA, with the factors “material”, “curing protocol”, and “aging condition”. As described above, significant factor interactions were followed by one-way ANOVAs to assess simple main effects, and multiple comparisons were corrected with Tukey’s adjustment. The statistical analysis was performed using SPSS Statistics, version 26.0 (IBM, Armonk, NY, USA), with the overall significance level set at α = 0.05.

## 3. Results

### 3.1. Polymerisation Kinetics and Degree of Conversion

**Figure 2:**
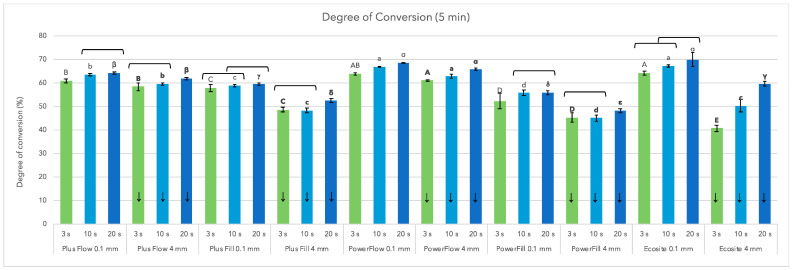
Degree of conversion of tested materials after 5 min, at the specimen thickness of 0.1 mm or 4 mm, using 3 s, 10 s, or 20 s curing. Down arrows denote significantly different groups in comparisons of 0.1 mm vs. 4 mm. The same letters denote statistically similar groups in comparisons among materials (uppercase, lowercase, and Greek letters for 3 s, 10 s, and 20 s, respectively); regular letters, within 0.1 mm; bold letters, within 4 mm. Square brackets denote no significant differences among curing protocols.

Figure 2 shows the DC values determined 5 min after the start of illumination near-surface (0.1 mm) and at a depth of 4 mm. As expected, the DC value was significantly higher at 0.1 mm than at 4 mm for all materials and for all curing protocols.

Rapid 3 s curing produced DC_5min_ values similar to those from 10 s curing for all AFCT materials, with differences ranging from 1 to 3%. However, only Plus Fill achieved statistically similar DC values for the 3 s and 10 s curing at 0.1 and 4 mm depth. PowerFlow and PowerFill achieved similar DC values for 3 s and 10 s curing only at 4 mm depth, while Ecosite surprisingly showed equally high DC values for 3 s vs. 10 s curing and 10 s vs. 20 s curing at the near-surface level of 0.1 mm.

The highest DC achieved Ecosite and PowerFlow at 0.1 mm (70.00 ± 3.07% and 68.05 ± 0.20%, respectively) at 20 s cure for both materials. However, Ecosite also had the lowest DC value at 4 mm depth (40.72 ± 1.46%) at 3 s curing, while PowerFlow had the highest DC value (62.02 ± 0.45%) under the same conditions. In general, for the DC values at 0.1 mm, Ecosite = PowerFlow > Plus Flow > Plus Fill > PowerFill, while at 4 mm and 3 s curing, the ranking was PowerFlow > Plus Flow > Plus Fill > PowerFill > Ecosite.

The reference material Ecosite was also the only one with a ratio of less than 80% between 4 mm and 0.1 mm DC at 3 s and 10 s curing. However, ratio over 80% was reached at 20 s curing at the same 4 mm depth (Table 2).

**Figure 3:**
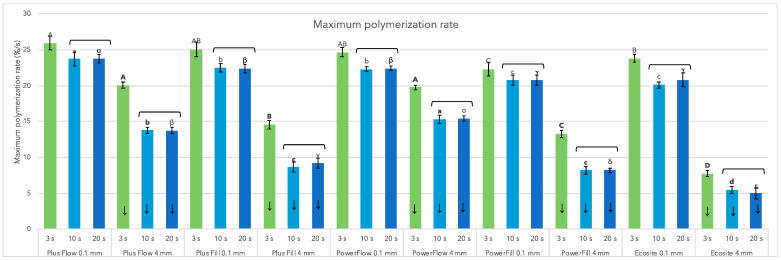
Maximum polymerisation rate (%/s) of tested materials, at the specimen thickness of 0.1 mm or 4 mm, using 3 s, 10 s, or 20 s curing. Down arrows denote significantly different groups in comparisons of 0.1 mm vs. 4 mm. The same letters denote statistically similar groups in comparisons among materials (uppercase, lowercase, and Greek letters for 3 s, 10 s, and 20 s, respectively); regular letters, within 0.1 mm; bold letters, within 4 mm. Square brackets denote no significant differences among curing protocols.

Similar to DC_5min_, the R_max_ (Figure 3) was always significantly lower at 4 mm depth than at 0.1 mm for all materials and curing conditions. The 3 s cure always showed the highest R_max_ regardless of the materials and depth, while the 10 s and 20 s curing showed statistically similar values.

**Figure 4:**
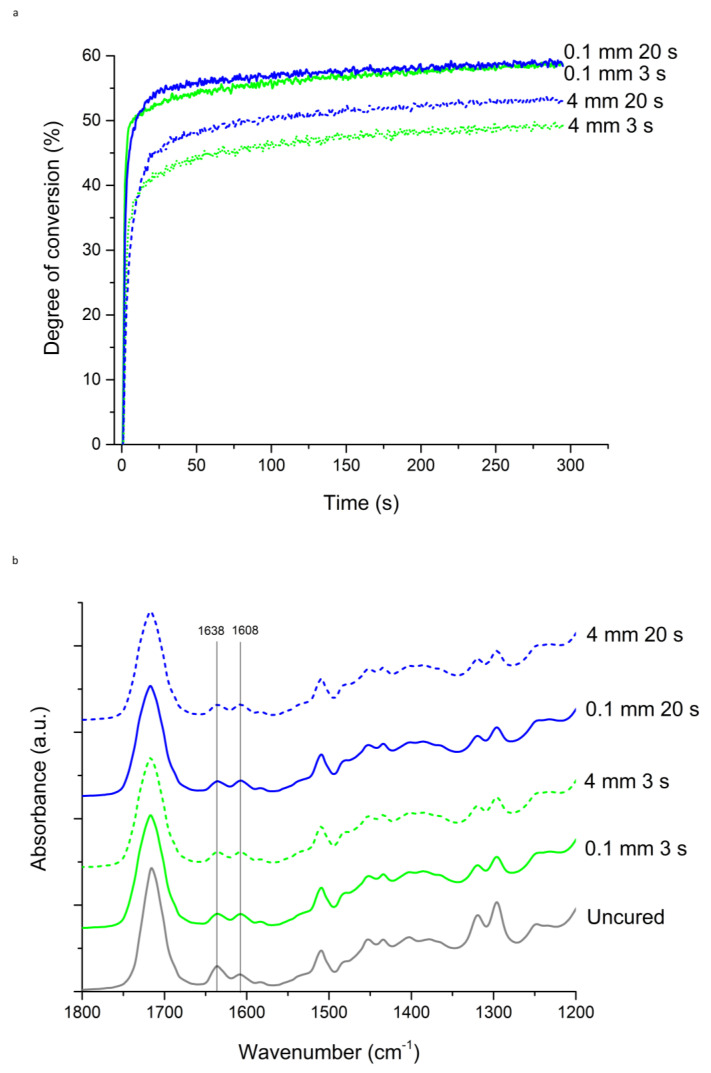
Polymerisation of Tetric Plus Fill material cured by 3 s or 20 s protocol at depths 0.1 or 4 mm: (**a**) time-dependent changes in the degree of conversion; (**b**) FTIR spectra of unpolymerised and polymerised samples.

Plus Flow, closely followed by Plus Fill and PowerFlow, showed the fastest polymerisation at the near-surface level (0.1 mm). The differences between the materials were more pronounced at a depth of 4 mm, where the flowable composites Plus Flow and PowerFlow showed the fastest polymerisation rate, while the sculptable materials showed slower R_max_ and ranked Plus Fill > PowerFill > Ecosite. Ecosite showed the slowest R_max_ at 4 mm depth and 20 s curing. Earlier start of polymerisation for 3 s curing in comparison to 20 s curing is depicted in Figure 4 for material Plus Fill.

As shown in Table 3, polymerisation kinetic parameters “a”, denoting the pre-gel phase of polymerisation, has the highest value for all the materials. Kinetic parameters “a” and “b”, both denoting the pre-gel phase, were higher for materials Plus Fill and Plus Flow for 3 s polymerisation than PowerFill and PowerFlow, respectively. At the same curing protocol, Plus Fill and Plus Flow had a lower “c” parameter, signifying the post-gel phase of polymerisation.

### 3.2. Flexural Properties

Under rapid 3 s curing, materials with the highest FS after 24 h were Ecosite and Plus Fill. TC significantly reduced the FS of most materials, whereas Ecosite remained the material with the highest FS and FM.

**Figure 5:**
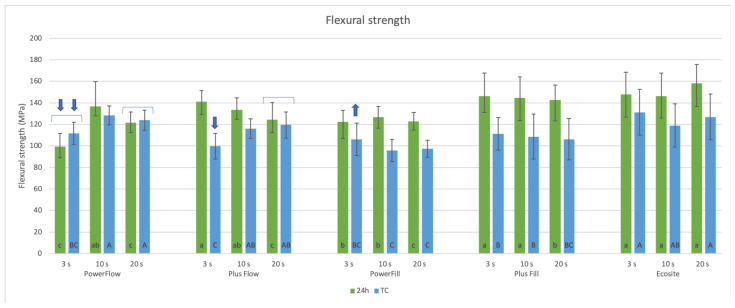
Flexural strength of tested materials cured with 3 s, 10 s, or 20 s protocol, after 24 h or thermal cycling (TC). Small letters: comparison of different materials with the same curing protocol at 24 h. Capital letters: comparison of different materials with the same curing protocol after thermal cycling. Brackets: no significant difference between 24 h and TC for the same material and curing protocol. Arrows: comparison of different curing protocols within each material, shown only for 3 s curing; 3 s curing is statistically lower (down arrows) or higher (up arrows) than 10 s curing.

When comparing the curing protocols for the same material (Figure 5), 3 s curing had no negative effect on the FS of the tested materials after 24 h, with the exception of the flowable material PowerFlow. In this group, Plus Flow showed a lower FS only at 20 s curing, while there was no difference between 3 s and 10 s. However, in the TC group, the flowable materials showed lower FS in the 3 s compared to 20 s curing, while the other materials showed no difference between 3 s, 10 s, and 20 s curing. The exception was PowerFill, which showed a similar FS at 3 s and 20 s curing, but a lower FS at 10 s curing.

A comparison of the different materials in terms of their FS performance after 24 h of water storage showed that 3 s and 10 s curing protocols did not change the ranking of the three materials with the highest FS: Plus Fill = Ecosite ≥ Plus Flow. Meanwhile, PowerFill and PowerFlow showed dependency on the curing. In the 20 s curing group at 24 h, Ecosite dominated as the material with the highest FS, followed by sculptable materials Plus Fill and PowerFill, while the flowable variants showed the lowest FS.

However, TC significantly reduced the FS of most materials (all except Plus Flow with 20 s and PowerFlow 3 s and 20 s curing). After TC, Ecosite was the material with the highest FS at 3 s curing, followed by Plus Fill ≥ PowerFill = PowerFlow ≥ Plus Flow. At 10 s and 20 s curing after TC, the ranking of the materials was the same: PowerFlow = Ecosite ≥ Plus Flow ≥ Plus Fill ≥ PowerFill, as shown in Figure 4.

Regardless of the curing protocol or ageing, all tested materials exhibited values of more than 80 MPa, which is required by ISO 4049 [35] for resin-based materials in load-bearing areas.

**Figure 6:**
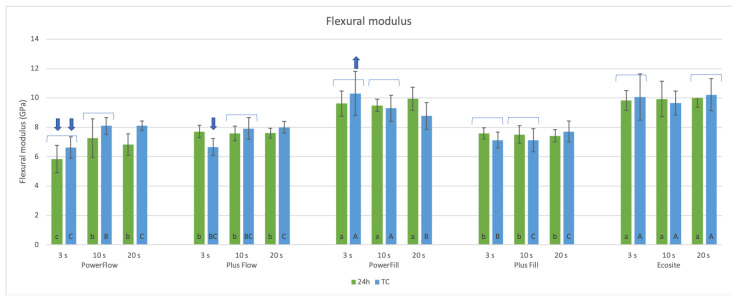
Flexural modulus of tested materials cured with 3 s, 10 s, or 20 s protocol, after 24 h or thermal cycling (TC). Small letters: comparison of different materials with the same curing protocol at 24 h. Capital letters: comparison of different materials with the same curing protocol after thermal cycling. Brackets: no significant difference between 24 h and TC for the same material and curing protocol. Arrows: comparison of different curing protocols within each material, shown only for 3 s curing; 3 s curing is statistically lower (down arrows) or higher (up arrows) than 10 s curing.

The PowerFill and Ecosite materials had the highest FM (Figure 6), both for 24 h and after TC, for all curing protocols except for the 20 s cure after TC, where Ecosite had a higher FM than PowerFill (Figure 5). TC resulted in different behaviour for the different materials: in some cases, the FM remained statistically similar (marked with brackets in Figure 5); in some cases, it increased (PowerFlow 20 s); and in the remaining cases, it decreased.

When comparing the influence of the curing protocols on the FM at the 24 h time point, there was no difference between the materials except for PowerFlow. However, after TC, the 3 s curing protocol statistically reduced FM for flowable materials and Plus Fill in comparison to 20 s. The only exceptions were Ecosite, which showed no difference between 3 s, 10 s, and 20 s curing, and PowerFill, which showed a higher FM at 3 s than at 10 s curing.

## 4. Discussion

The present study investigated the polymerisation efficiency and flexural properties of a new class of universal bulk-fill composites, Plus Fill and Plus Flow, specifically developed for anterior and posterior applications in 4 mm increments. These materials should be able to overcome the traditional limitations of the previous generation of bulk-fill composites, namely excessive translucency and insufficient aesthetic integration in the anterior region, while remaining mechanically reliable even under highly accelerated polymerisation conditions [7].

The first hypothesis is accepted, because new universal composites showed differences in DC, FS, and FM compared to their predecessors and non-AFCT reference material. Plus Fill was the only material that had no negative consequence of 3 s curing for any of the tested properties, DC at both depths, and FS and FM before and after TC. The second hypothesis was partially rejected, as the results show that the 3 s curing caused a lower DC than 10 s curing at both depths, at the near surface (0.1 mm) and at 4 mm depth, for all the materials, except for Plus Fill. The accelerated aging by TC negatively affected the FS of all the tested materials in all curing conditions except for Plus Flow and PowerFlow cured by 20 s protocol and PowerFlow cured by 3 s protocol.

### 4.1. Polymerisation Behaviour

This study investigated early-stage polymerisation behaviour. Although post-cure reactions continue for up to 24 h [39,40], the initial seconds after light exposure determine the spatiotemporal development of radical populations and, consequently, the depth-dependent conversion profile [41,42].

All materials and curing protocols showed the expected decline in DC at 4 mm depth. This result could be explained by the exponential light attenuation once vitrification in the surface layers alters refractive index of the resin and restricts both photon transport and radical diffusion [42,43,44]. This depth effect was evident in the markedly higher pre-gel parameter “a” at 0.1 mm than at 4 mm, whereas the post-gel parameter “c” showed the opposite trend. Flowable materials PowerFlow and Plus Flow had the lowest filler volume, so the light attenuation was the lowest for them, showing the highest bottom/top DC ratio.

Under 3 s high-irradiance curing, the rapid radical burst promotes early bimolecular termination and accelerated vitrification in conventional resin composites. This is consistent with known deviations from exposure reciprocity law [41,45,46], especially in flowable composites [47]. As polymerisation progressed, increased light scattering in the polymerised material further limited light transmission. This later-stage network development seems to be the principal determinant of DC in deeper layers [27,41], not only the radiant exposure. Hence, the 20 s protocol produced the highest DC_5min_ at 4 mm.

Nonetheless, all RAFT-based materials maintained clinically acceptable bottom-to-top DC ratios under both 3 s and 10 s curing. Their performance can be attributed to the combination of an AFCT reagent and the germanium-based initiator Ivocerin, whose high molar extinction coefficient and spectral overlap with the LCU’s violet emission (≈410 nm), ensuring efficient initiation at depth and sustained radical activity during post-cure. Therefore, early photon transport through the composite is enhanced during the first seconds of cure [27]. Extended radical activity supported by RAFT fragmentation, combined with the high initiation efficiency of Ivocerin, enables DC at depth to continue rising during the post-cure phase. In contrast, Ecosite, as the non-AFCT reference material, did not reach the 80% bottom-to-top ratios for 3 s and 10 s curing, which is not surprising since the manufacturer-recommended curing time is 20 s with >1000 mW/cm^2^.

The comparison between the new universal RAFT-modified materials (Plus Fill and Plus Flow) and their predecessors (PowerFill and PowerFlow) shows clear differences in early radical dynamics and depth-dependent polymerisation behaviour. In the pre-gel phase, as indicated by kinetic parameters “a” and “b”, both Plus materials showed higher values than their corresponding Power predecessors under 3 s polymerisation, indicating a more rapid initial conversion and greater early radical flux. This accelerated onset was followed by comparatively lower “c” values in the post-gel phase, suggesting that the later stages of network formation progressed more slowly in the Plus materials than in PowerFill and PowerFlow under identical curing conditions. Similar results were obtained by Ilie for almost identical materials, but at 2 and 4 mm depth [10]. In the subsurface (0.1 mm), polymerisation proceeded fastest in Plus Flow, followed by Plus Fill and PowerFlow. At 4 mm, the flowable materials (Plus Flow and PowerFlow) again showed the highest R_max_, with sculptable materials ranking as Plus Fill > PowerFill > Ecosite. Ecosite consistently polymerised slowest at depth, particularly under 20 s curing. Only Plus Fill achieved equivalent DC for 3 s and 10 s curing at both depths, indicating the highest efficiency of curing at depth among all tested materials. Thus, the new Tetric Plus composites accelerate early kinetic phases without compromising deep polymerisation, thus confirming their suitability for ultrafast, high-irradiance curing.

### 4.2. Mechanical Performance and Effect of Ageing

Accelerated ageing protocols are essential for predicting the long-term clinical behaviour of resin composites, as they reproduce, in a compressed timeframe, the hydrolytic and thermo-mechanical stresses that are present intraorally [8,9]. In this study, seven-day water storage initiated the well-documented early degradation processes: polymer plasticisation, leaching of unreacted species, and initial hydrolysis of the filler–matrix interface [48]. Subsequent exposure to 10,000 thermal cycles between 5 °C and 55 °C introduced repeated contraction/expansion stresses resulting from mismatched thermal expansion coefficients of the polymer matrix and inorganic fillers, promoting interfacial debonding, microcrack propagation, and cumulative structural fatigue [49]. This combined protocol is among the few ageing methods that correlate well with in vivo studies [33,34,50,51]. In the present study, there was an expected decline in FS for all materials tested, except for flowable materials cured with the 20 s protocol, and PowerFlow cured with the 3 s protocol, which also had the lowest FS. Nevertheless, all FS values were at least 10 MPa higher than the ISO 4049 requirements, even after TC [35].

The results of this study showed no direct association between DC and mechanical performance. Ecosite achieved the highest FS and FM at both 24 h and after ageing, yet its DC at 4 mm was only mid-range. On the other hand, flowable composites such as PowerFlow and Plus Flow displayed high DC but were more strongly affected by curing mode and thermal cycling. These findings emphasise that mechanical properties depend not only on DC but also on the organisation and resilience of the polymer network formed during curing [42,52,53,54]. However, the filler content and silanisation at the filler–resin interface remain among the most important determinants of mechanical properties [8,55,56]. This is why Ecosite, as the most highly filled material, retained the highest FS and FM, even when cured with the 3 s protocol, which was not recommended by the manufacturer. Similarly, the flowable composites had higher resin content, which could explain their greater susceptibility to hydrolytic degradation [57].

A substantial reduction in FS and FM after thermal cycling is observed in the flowable composites cured for only 3 s at ≈3000 mW/cm^2^ compared to 10 s and 20 s curing for the same material. It is important to emphasise that the energy delivered to the specimens during 3 s curing was less than half that delivered by the 20 s protocol, and even 25% lower than the energy delivered by the 10 s protocol. Without measurement of the actual crosslinking density and full disclosure of the materials’ content, we can only hypothesise the reasons behind these results. Although these materials reached high DC, such short exposures to high energy tend to generate extremely high radical concentrations that accelerate gelation and cause early vitrification [27,45]. This could limit the capacity of AFCT reagent to homogenise the forming network. The resulting polymer likely comprises highly converted but irregularly crosslinked domains with elevated internal stress and increased free volume [42].

During water storage, these heterogeneous crosslink regions absorb more water, become plasticised, and weaken the filler/matrix interface [9,58,59]. This effect was seen in our previous study on PowerFlow cured by 3 s protocol, which showed more significant FS degradation in water at 2–4 mm depth [16]. Klaric et al. also found higher solubility of composites cured by 3 s protocol [59]. Thermal cycling further exacerbates the damage, amplifying pre-existing stress and promoting microcrack growth [48]. In contrast, the 10 and 20 s curing conditions extend radical mobility and allow AFCT reactions to more effectively redistribute stress and form a more uniform, fatigue-resistant structure [11,60]. Flowable materials, with lower filler content and higher resin fraction, are inherently more vulnerable to hydrolytic degradation than sculptable materials [48,51,61,62,63]. Furthermore, network irregularities are more detrimental when inorganic content is low [47,48]. In sculptable materials, the higher filler content and lower resin fraction reduce the probability of rapid bimolecular termination and make their mechanical behaviour less sensitive to curing protocol [47], consistent with the present results.

The relationship between filler volume and FM was confirmed, with the ranking Ecosite > PowerFill > Plus Fill > Plus Flow > PowerFlow matching their respective filler contents. Similar findings were found in a study by Ilie; however, the curing parameters used in that study were 20 s and ~1400 mW/cm^2^ [29]. As described by Fu et al. [56], filler volume is a primary determinant of stiffness, overshadowing differences in silanisation or DC. Thus, flowable composites with low filler amounts had high conversion but the lowest FM under all conditions.

One of the limitations of this study is the difference in curing of DC and mechanical tests. DC was assessed after a single curing exposure to the top surface, whereas the flexural specimens received six curing cycles (three per side), resulting in substantially higher total radiant exposure. Despite this, the observations and conclusion for each of the tests individually remained valid. While TC provides a meaningful accelerated ageing model, it does not reproduce all intraoral challenges. Future research should incorporate long-term storage, fatigue loading, and validation in clinical trials [64]. Investigating the influence of cavity geometry and clinically relevant light-incidence angles would further refine the understanding of bulk-fill composite behaviour.

## 5. Conclusions

Within the limitations of this in vitro study, the following conclusions can be drawn:

1. New universal Tetric Plus composites accelerate early polymerisation without compromising deep polymerisation.

2. The suitability of rapid curing depends on the material and is not universally reliable for non-AFCT composites.

3. All tested materials, under tested curing protocols and aging conditions, satisfied the ISO 4049 requirements for flexural strength.

4. For tested flowable composites, 3 s curing led to lower resistance to hydrothermal ageing and therefore to inferior mechanical performance.

5. Longer curing times (10–20 s) are advisable, particularly for flowable materials, in deeper cavities, and in clinical situations where optimal light delivery cannot be ensured.

## Figures and Tables

**Table 1:** Materials used in the study and their composition according to the manufacturers.

Materials (Shade, Manufacturer)	Consistency	Resin	Fillers
**Tetric Plus Flow** **(A2; Ivoclar)**	Flowable	Bis-GMA, Bis-EMA, UDMA, BPEMA, MOMA, DCP	65 wt%/ 50–51 vol%
**Tetric Plus Fill** **(A2; Ivoclar)**	Sculptable	Bis-GMA, Bis-EMA, UDMA, Aromatic-Aliphatic UDMA, DCP	70 wt%/ 51–52 vol%
**Tetric PowerFlow (IVA; Ivoclar)**	Flowable	Bis-GMA, Bis-EMA, UDMA	68.2 wt%/ 46.4 vol%
**Tetric PowerFill (IVA; Ivoclar)**	Sculptable	Bis-GMA, Bis-EMA, UDMA, propoxylated bisphenol A dimethacrylate, DCP	77 wt%/ 54 vol%
**Reference material—Ecosite Bulk Fill (universal; DMG, Hamburg, Germany)**	Sculptable	Bis-EMA, SiO_2_, bis-GMA, NPG2PODA, UDMA, additives	85 wt%/ 65 vol%

Bis-GMA, bisphenol A glycidyl methacrylate; Bis-EMA, ethoxylated bisphenol A dimethacrylate; UDMA, urethane dimethacrylate and aromatic dimethacrylate; BPEMA, ethoxylated biphenyl methacrylate; MOMA, methacryloyloxyethyl urethane methacrylate; DCP, tricyclodecane-dimethanol dimethacrylate;; NPG2PODA, neopentyl glycol propoxylate diacrylate.

**Table 2:** Botton/top ratios of mean DC values after 5 min at 4 mm and 0.1 mm (%).

	3 s	10 s	20 s
Plus Flow	95.78	93.91	96.41
Plus Fill	84.31	81.96	88.09
PowerFlow	95.61	93.63	95.96
PowerFill	86.69	80.90	86.19
Ecosite	63.36	74.83	85.07

**Table 3:** The calculated a, b, c, and d parameters for the tested materials, curing protocols (3 s, 10 s, and 20 s), and depths (0.1 and 4 mm).

	a				b				c				d			
Group	Value	StEr	LB	UB	Value	StEr	LB	UB	Value	StEr	LB	UB	Value	StEr	LB	UB
Plus Flow 0.1 mm 3 s	85.75	0.85	82.03	89.47	0.72	0.01	0.68	0.76	8.21	0.13	7.65	8.78	0.01	0.00	0.01	0.01
Plus Flow 0.1 mm 10 s	78.91	0.56	76.44	81.38	0.59	0.01	0.56	0.61	7.00	0.07	6.69	7.31	0.01	0.00	0.01	0.01
T Plus Flow 0.1 mm 20 s	80.21	0.71	77.09	83.34	0.52	0.01	0.50	0.55	6.59	0.11	6.09	7.09	0.01	0.00	0.01	0.02
Plus Flow 4 mm 3 s	71.26	0.69	68.23	74.29	0.55	0.01	0.52	0.58	10.18	0.10	9.75	10.62	0.01	0.00	0.01	0.01
Plus Flow 4 mm 10 s	64.48	0.43	62.59	66.36	0.29	0.00	0.28	0.30	9.55	0.10	9.10	10.00	0.01	0.00	0.01	0.01
Plus Flow 4 mm 20 s	63.11	0.39	61.42	64.80	0.31	0.00	0.29	0.32	9.61	0.11	9.13	10.08	0.01	0.00	0.01	0.02
Plus FIll 0.1 mm 3 s	71.85	1.01	67.41	76.30	0.58	0.01	0.54	0.63	5.50	0.12	4.96	6.04	0.01	0.00	0.01	0.01
Plus FIll 0.1 mm 10 s	72.17	0.79	68.69	75.65	0.49	0.01	0.45	0.52	7.41	0.12	6.90	7.91	0.01	0.00	0.01	0.01
Plus FIll 0.1 mm 20 s	70.79	0.78	67.38	74.20	0.52	0.01	0.48	0.55	7.76	0.14	7.14	8.38	0.02	0.00	0.01	0.02
Plus FIll 4 mm 3 s	53.29	0.67	50.33	56.24	0.38	0.01	0.34	0.41	10.09	0.14	9.47	10.71	0.01	0.00	0.01	0.02
Plus FIll 4 mm 10 s	46.02	0.35	44.47	47.58	0.24	0.00	0.22	0.25	10.32	0.10	9.86	10.77	0.01	0.00	0.01	0.01
Plus FIll 4 mm 20 s	52.69	0.35	51.14	54.23	0.17	0.00	0.16	0.17	8.96	0.13	8.37	9.55	0.01	0.00	0.01	0.01
Powerflow 0.1 mm 3 s	83.72	0.83	80.11	87.34	0.68	0.01	0.64	0.72	9.37	0.09	8.99	9.75	0.01	0.00	0.01	0.01
Powerflow 0.1 mm 10 s	79.68	0.63	76.91	82.45	0.41	0.00	0.39	0.43	8.24	0.10	7.79	8.70	0.01	0.00	0.01	0.01
Powerflow 0.1 mm 20 s	78.37	0.63	75.59	81.15	0.41	0.00	0.38	0.43	7.72	0.12	7.20	8.24	0.01	0.00	0.01	0.01
Powerflow 4 mm 3 s	69.08	0.69	66.06	72.09	0.54	0.01	0.50	0.57	12.21	0.10	11.78	12.64	0.01	0.00	0.01	0.01
Powerflow 4 mm 10 s	64.17	0.39	62.45	65.90	0.33	0.00	0.31	0.34	11.68	0.09	11.29	12.06	0.01	0.00	0.01	0.01
Powerflow 4 mm 20 s	65.09	0.48	62.99	67.18	0.27	0.00	0.26	0.29	11.19	0.14	10.57	11.82	0.01	0.00	0.01	0.02
Powerfill 0.1 mm 3 s	67.71	0.99	63.34	72.07	0.61	0.01	0.56	0.66	6.96	0.12	6.45	7.47	0.01	0.00	0.01	0.01
Powerfill 0.1 mm 10 s	65.22	0.69	62.19	68.26	0.49	0.01	0.46	0.52	8.42	0.10	8.00	8.85	0.01	0.00	0.01	0.01
Powerfill 0.1 mm 20 s	66.16	0.77	62.80	69.51	0.45	0.01	0.41	0.48	7.40	0.15	6.75	8.04	0.01	0.00	0.01	0.02
Powerfill 4 mm 3 s	49.83	0.54	47.47	52.18	0.43	0.01	0.40	0.46	11.14	0.10	10.72	11.55	0.01	0.00	0.01	0.01
Powerfill 4 mm 10 s	46.05	0.32	44.63	47.46	0.25	0.00	0.23	0.26	9.80	0.09	9.40	10.21	0.01	0.00	0.01	0.01
Powerfill 4 mm 20 s	43.23	0.26	42.09	44.38	0.19	0.00	0.18	0.20	9.79	0.09	9.38	10.19	0.01	0.00	0.01	0.01
Ecosite 0.1 mm 3 s	83.54	1.12	78.65	88.44	0.47	0.01	0.43	0.51	9.08	0.16	8.36	9.80	0.01	0.00	0.01	0.01
Ecosite 0.1 mm 10 s	80.15	0.61	77.46	82.85	0.38	0.00	0.36	0.40	8.07	0.11	7.59	8.55	0.01	0.00	0.01	0.01
Ecosite 0.1 mm 20 s	87.72	0.76	84.38	91.07	0.35	0.00	0.33	0.37	8.90	0.16	8.20	9.59	0.01	0.00	0.01	0.01
Ecosite 4 mm 3 s	35.15	0.44	33.22	37.07	0.24	0.01	0.22	0.27	13.72	0.11	13.23	14.22	0.01	0.00	0.01	0.01
Ecosite 4 mm 10 s	52.08	0.42	50.23	53.92	0.15	0.00	0.14	0.16	13.10	0.15	12.45	13.76	0.01	0.00	0.01	0.01
Ecosite 4 mm 20 s	58.02	0.24	56.97	59.06	0.13	0.00	0.13	0.14	10.67	0.10	10.25	11.09	0.01	0.00	0.01	0.01

StEr, standard error; LB, lower bound of the 95% confidence interval; UB, upper bound of the 95% confidence interval.

## Data Availability

The original contributions presented in this study are included in the article. Further inquiries can be directed to the corresponding author.
